# Trends in Deaths Involving Heroin and Synthetic Opioids Excluding Methadone, and Law Enforcement Drug Product Reports, by Census Region — United States, 2006–2015

**DOI:** 10.15585/mmwr.mm6634a2

**Published:** 2017-09-01

**Authors:** Julie K. O’Donnell, R. Matthew Gladden, Puja Seth

**Affiliations:** 1 Division of Unintentional Injury Prevention, National Center for Injury Prevention and Control, CDC.

Opioid overdose deaths quadrupled from 8,050 in 1999 to 33,091 in 2015 and accounted for 63% of drug overdose deaths in the United States in 2015. During 2010–2015, heroin overdose deaths quadrupled from 3,036 to 12,989 (*1*). Sharp increases in the supply of heroin and illicitly manufactured fentanyl (IMF) are likely contributing to increased deaths (*2*–*6*). CDC examined trends in unintentional and undetermined deaths involving heroin or synthetic opioids excluding methadone (i.e., synthetic opioids)* by the four U.S. Census regions during 2006–2015. Drug exhibits (i.e., drug products) obtained by law enforcement and reported to the Drug Enforcement Administration’s (DEA’s) National Forensic Laboratory Information System (NFLIS) that tested positive for heroin or fentanyl (i.e., drug reports) also were examined. All U.S. Census regions experienced substantial increases in deaths involving heroin from 2006 to 2015. Since 2010, the South and West experienced increases in heroin drug reports, whereas the Northeast and Midwest experienced steady increases during 2006–2015.^†^ In the Northeast, Midwest, and South, deaths involving synthetic opioids and fentanyl drug reports increased considerably after 2013. These broad changes in the U.S. illicit drug market highlight the urgent need to track illicit drugs and enhance public health interventions targeting persons using or at high risk for using heroin or IMF.

Full-year estimates of fentanyl or heroin drug reports^§^ per 100,000 population (using DEA’s NFLIS)^¶^ and unintentional or undetermined intent heroin and synthetic opioid death rates per 100,000 population (using the National Vital Statistics System multiple cause-of-death mortality files) were stratified by the four U.S. Census regions for 2006–2015. The following *International Classification of Diseases, Tenth Revision* codes were used to identify deaths involving heroin and synthetic opioids: 1) underlying cause-of-death codes X40–X44 (unintentional) or Y10–Y14 (undetermined)** and 2) opioid-specific multiple cause-of-death codes of T40.1 for heroin and T40.4 for synthetic opioids. Total deaths involving heroin (i.e., deaths involving heroin with or without synthetic opioids) and total deaths involving synthetic opioids (i.e., deaths involving synthetic opioids with or without heroin) were categorized further into three groups: 1) deaths involving heroin without synthetic opioids, 2) deaths involving synthetic opioids without heroin, and 3) deaths involving use of both heroin and synthetic opioids.^††^ Changes in deaths involving synthetic opioids after 2013 have been primarily driven by IMF and thus are a proxy for changes in deaths involving fentanyl after 2013 (*5*,*6*).^§§^ Piecewise linear regression analyses were used to examine hypotheses that regional trends mirrored the national trends in which deaths involving heroin and heroin drug reports increased at faster rates starting in 2010 and deaths involving synthetic opioids and fentanyl drug reports increased at faster rates starting in 2013. To examine the impact of synthetic opioids on the increase in deaths involving heroin without synthetic opioids, rate increases in deaths involving heroin without synthetic opioids were examined before and after 2013.

The rate of deaths involving heroin increased during 2006–2015 nationally and in all four U.S. Census regions (Table). Total deaths involving heroin increased more sharply during 2010–2015 than during 2006–2009 in all regions, with the largest increases occurring during 2010–2015 in the Northeast (average yearly increase of 1.02 deaths per 100,000 population) and Midwest (average yearly increase of 0.89 deaths per 100,000 population) (Table). Heroin drug report trends mirrored trends in total deaths involving heroin, with overall increases during 2006–2015 in all regions. The South and West experienced larger average yearly increases in rates of heroin drug reports during 2010–2015 than during 2006–2009 (Table). In contrast, the Northeast and Midwest experienced steady increases in rates of heroin drug reports during 2006–2015 (Figure 1).

**TABLE:** Average yearly changes in rates of overdose deaths involving heroin and synthetic opioids excluding methadone, and law enforcement drug reports of heroin and fentanyl, by census region — United States, 2006–2015

Event, period	National	U.S. Census region*			
		Northeast	Midwest	South	West
	Rate change (95% CI)	Rate change (95% CI)	Rate change (95% CI)	Rate change (95% CI)	Rate change (95% CI)
**Total deaths involving heroin**					
2006–2009	0.13 (0.01–0.26)^†^	0.11 (-0.04–0.27)	0.26 (0.00–0.53)^†^	0.08 (-0.10–0.26)	0.10 (0.02–0.18)^†^
2010–2015	0.62 (0.55–0.68)^†,§^	1.02 (0.93–1.10)^†,§^	0.89 (0.74–1.03)^†,§^	0.49 (0.39–0.59)^†,§^	0.28 (0.24–0.33)^†,§^
**Heroin drug reports** ^¶^					
2006–2009	2.24 (0.51–3.96)^†^	4.49 (-0.55–9.52)	4.88 (1.46–8.30)^†^	0.24 (-0.77–1.26)	1.40 (-0.09–2.88)
2010–2015	4.52 (3.60–5.44)^†,§^	8.51 (5.81–11.20)^†^	6.00 (4.17–7.83)^†^	2.74 (2.20–3.28)^†,§^	3.33 (2.53–4.12)^†,§^
**Total deaths involving synthetic opioids**					
2006–2012	0.01 (-0.05–0.06)	0.02 (-0.08–0.12)	-0.06 (-0.18–0.07)	0.03 (-0.02–0.08)	0.02 (-0.01–0.06)
2013–2015	0.98 (0.78–1.18)^†,§^	2.15 (1.77–2.52)^†,§^	1.35 (0.88–1.81)^†,§^	0.81 (0.63–0.98)^†,§^	0.07 (-0.07–0.20)
**Fentanyl drug reports** ^¶^					
2006–2012	-0.10 (-0.29–0.08)	-0.20 (-0.64–0.24)	-0.31 (-0.75–0.13)	-0.002 (-0.053–0.049)	0.017 (-0.002–0.037)
2013–2015	2.09 (1.40–2.79)^†,§^	5.08 (3.42–6.75)^†,§^	3.64 (1.99–5.29)^†,§^	1.08 (0.89–1.27)^†,§^	0.11 (0.03–0.18)^†,§^
**Deaths involving heroin without synthetic opioids**					
2006–2012	0.17 (0.09–0.25)^†^	0.26 (0.07–0.44)^†^	0.33 (0.25–0.41)^†^	0.08 (0.02–0.14)^†^	0.10 (0.04–0.15)^†^
2013–2015	0.33 (0.03–0.62)^†^	0.31 (-0.37–0.99)	0.23 (-0.07–0.53)	0.43 (0.20–0.67)^†,§^	0.27 (0.05–0.49)^†^
**Deaths involving synthetic opioids without heroin**					
2006–2012	0.01 (-0.03–0.05)	0.02 (-0.05–0.08)	-0.05 (-0.14–0.04)	0.03 (-0.01–0.07)	0.02 (-0.01–0.06)
2013–2015	0.60 (0.45–0.75)^†,§^	1.31 (1.07–1.55)^†,§^	0.73 (0.38–1.07)^†,§^	0.54 (0.38–0.69)^†,§^	0.05 (-0.08–0.19)
**Deaths involving use of both heroin and synthetic opioids**					
2006–2012	-0.001 (-0.021–0.020)	0.001 (-0.037–0.039)	-0.008 (-0.055–0.040)	0.002 (-0.010–0.013)	0.001 (-0.001–0.003)
2013–2015	0.384 (0.307–0.460)^†,§^	0.842 (0.701–0.982)^†,§^	0.622 (0.444–0.800)^†,§^	0.269 (0.228–0.311)^†,§^	0.014 (0.005–0.023)^†,§^

**Abbreviation:** CI = confidence interval.

* *Northeast:* Connecticut, Maine, Massachusetts, New Hampshire, New Jersey, New York, Pennsylvania, Rhode Island, and Vermont; *Midwest:* Illinois, Indiana, Iowa, Kansas, Michigan, Minnesota, Missouri, Nebraska, North Dakota, Ohio, South Dakota, and Wisconsin; *South:* Alabama, Arkansas, Delaware, District of Columbia, Florida, Georgia, Kentucky, Louisiana, Maryland, Mississippi, North Carolina, Oklahoma, South Carolina, Tennessee, Texas, Virginia, and West Virginia; *West:* Alaska, Arizona, California, Colorado, Hawaii, Idaho, Montana, Nevada, New Mexico, Oregon, Utah, Washington, and Wyoming.

^†^ Slope estimates are statistically significantly different from zero (p<0.05).

^§^ Slope for the later period is statistically significantly different from that of the earlier period (p<0.05).

^¶^ Drug exhibits (i.e., drug products) obtained by law enforcement that tested positive for heroin or fentanyl and were reported to the Drug Enforcement Administration’s National Forensic Laboratory Information System.

**FIGURE 1:**
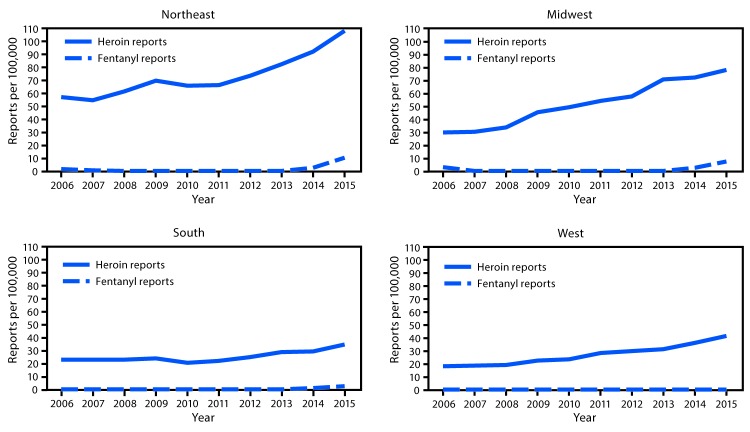
Number of law enforcement drug reports for heroin and fentanyl per 100,000 population, by census region* — United States, 2006–2015 * *Northeast:* Connecticut, Maine, Massachusetts, New Hampshire, New Jersey, New York, Pennsylvania, Rhode Island, and Vermont; *Midwest:* Illinois, Indiana, Iowa, Kansas, Michigan, Minnesota, Missouri, Nebraska, North Dakota, Ohio, South Dakota, and Wisconsin; *South:* Alabama, Arkansas, Delaware, District of Columbia, Florida, Georgia, Kentucky, Louisiana, Maryland, Mississippi, North Carolina, Oklahoma, South Carolina, Tennessee, Texas, Virginia, and West Virginia; *West:* Alaska, Arizona, California, Colorado, Hawaii, Idaho, Montana, Nevada, New Mexico, Oregon, Utah, Washington, and Wyoming.

During 2013–2015, deaths involving synthetic opioids without heroin increased at faster rates than did deaths involving heroin without synthetic opioids in the Northeast, Midwest, and South (Table) (Figure 2). Deaths involving use of both heroin and synthetic opioids had larger average yearly rate increases after 2013 than did deaths involving heroin without synthetic opioids in the Northeast (0.84 compared with 0.31 per 100,000 population) and Midwest (0.62 compared with 0.23), the two regions that experienced the largest increases in total deaths involving heroin (Table). Mirroring the pattern in deaths involving synthetic opioids, rates of fentanyl drug reports increased significantly nationally and across all regions starting in 2013 after having remained level during 2006–2012 (Figure 1).

**FIGURE 2:**
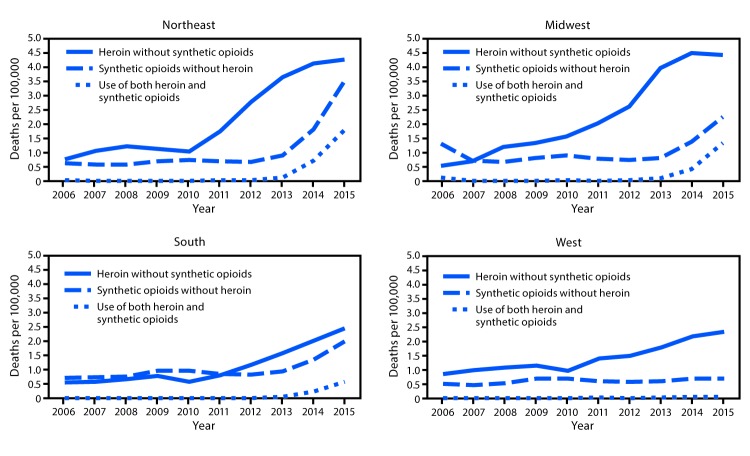
Number of deaths per 100,000 population involving heroin without synthetic opioids, synthetic opioids without heroin, and use of both heroin and synthetic opioids, by census region* — United States, 2006–2015 * *Northeast:* Connecticut, Maine, Massachusetts, New Hampshire, New Jersey, New York, Pennsylvania, Rhode Island, and Vermont; *Midwest:* Illinois, Indiana, Iowa, Kansas, Michigan, Minnesota, Missouri, Nebraska, North Dakota, Ohio, South Dakota, and Wisconsin; *South:* Alabama, Arkansas, Delaware, District of Columbia, Florida, Georgia, Kentucky, Louisiana, Maryland, Mississippi, North Carolina, Oklahoma, South Carolina, Tennessee, Texas, Virginia, and West Virginia; *West:* Alaska, Arizona, California, Colorado, Hawaii, Idaho, Montana, Nevada, New Mexico, Oregon, Utah, Washington, and Wyoming.

## Discussion

Three interconnected trends drove increases in unintentional or undetermined deaths involving heroin and synthetic opioids in the United States during 2006–2015: increases in the supply and use of heroin (*3*), mixing of fentanyl into the heroin supply, and increases in deaths involving synthetic opioids without heroin. Large increases in the heroin supply coincided with increases in deaths involving heroin across all U.S. Census regions, with the largest increases in the Northeast and Midwest. In 2016, many state and local law enforcement agencies in these regions reported that heroin could be easily obtained in their communities and was the top drug threat (*2*). The increasing availability of heroin comes at a time when an estimated 2 million persons reported a substance use disorder involving misuse of prescription opioids and nearly 600,000 reported a substance use disorder involving heroin in 2015.^¶¶^ Increased heroin availability combined with high potency and relatively low price might have made heroin a viable substitute because its effects are similar to those of prescription opioids (*7*). The strongest risk factor for heroin use and dependence is misuse of or dependence on prescription opioids; approximately 75% of persons who initiate heroin first misused prescription opioids (*7*), although only a small percentage of persons misusing prescription opioids begin using heroin.***

The second trend contributing to increases in deaths involving heroin and synthetic opioids is the mixing of fentanyl into the heroin supply by drug traffickers and persons misusing opioids. In 2015, DEA and CDC released a nationwide alert and a public health advisory^†††^ about large increases in fentanyl drug reports and deaths across multiple states in the Northeast, Midwest, and South, beginning in late 2013 (*8*). The high potency and rapid onset of action of fentanyl and fentanyl analogs and the difficulty of mixing nonlethal doses makes fentanyl more dangerous to use than heroin (*8*). Approximately half of the increase in deaths involving heroin after 2013 is attributable to increases in deaths involving use of both heroin and fentanyl. In the Northeast and Midwest, the U.S regions reporting the sharpest increases in fentanyl drug reports, deaths involving use of both heroin and synthetic opioids accounted for 77% of the total increase in deaths involving heroin. One possible reason for the relative stability of total deaths involving synthetic opioids and fentanyl drug reports in the West is that the form of heroin sold primarily west of the Mississippi River (black tar heroin) is difficult to mix with fentanyl, whereas white powder heroin, the type primarily sold east of the Mississippi, is more easily mixed with fentanyl (*2*). Reports from 14 states^§§§^ have shown that increases in deaths involving fentanyl (from 2,418 in 2014 to 4,980 in 2015) accounted for most of the increase in deaths involving synthetic opioids (from 2,658 in 2014 to 4,806 in 2015). These data, coupled with recent public health and law enforcement reports, continue to demonstrate that increases in deaths involving synthetic opioids are primarily driven by IMF (*2*,*5*,*6*).

Finally, multiple factors are likely driving the substantial increases in deaths involving synthetic opioids without heroin after 2013. First, the difficulty in distinguishing deaths involving morphine from deaths involving heroin might result in misclassifying some deaths as involving synthetic opioids without heroin (*9*). Also, drug products containing IMF are rapidly evolving, with IMF distributed in counterfeit prescription pills (*4*), mixed with and sold as cocaine, or sold as powders to persons using heroin with and without their knowledge that the product contains fentanyl (*2*,*6*).

The findings in this report are subject to at least five limitations. First, deaths involving heroin and synthetic opioids are likely underestimated because 17% of death certificates for drug overdose deaths lack information on the specific drug(s) involved, and this percentage varies widely by state (*1*). Second, although analyses excluded intentional deaths, it is possible that deaths involving heroin and synthetic opioids of undetermined intent might include homicides or suicides; however, only 7% of the analyzed deaths were categorized as undetermined. Third, toxicologic testing for synthetic opioids might have increased during 2006–2015, leading to higher rates because of better detection. Fourth, deaths involving synthetic opioids were a proxy for change in deaths involving fentanyl, but deaths involving other synthetic opioids, like tramadol, were included, which might result in underestimating increases in deaths involving fentanyl. Finally, NFLIS drug reports might vary because of jurisdictional differences in drug evidence submission and testing practices.

The heroin and IMF drug market in the United States is rapidly expanding in the context of widespread prescription opioid misuse. As a result, opioid-involved deaths are currently at peak reported levels. Enhanced, timely surveillance of the illicit drug supply and opioid overdoses is critical to rapidly track and respond to broad changes in the illicit opioid drug market, including the introduction of fentanyl analogs. Comprehensive testing of opioid overdose deaths is needed because fentanyl analogs, including potent analogs such as carfentanil, are increasingly distributed in illicit markets^¶¶¶^ (*10*). Evidence-based interventions such as implementing safer opioid prescribing,**** increasing naloxone availability,^††††^ and linking persons at high risk for an opioid overdose (e.g., persons treated in the emergency department for an overdose or persons released from prison, with a history of substance use disorder^§§§§^) to medication-assisted treatment, as well as geographically tailored responses linking law enforcement and public health, could help reduce opioid-involved morbidity and mortality. Finally, community-based services, like syringe exchange programs, can be leveraged to prevent infectious disease transmission among persons who inject drugs and as opportunities to connect persons with substance use disorders into care, risk-reduction services, and long-term recovery.

**Summary**

**What is already known about this topic?**

Opioid overdose deaths in the United States have been increasing since 1999, initially driven by prescription opioid misuse and more recently by heroin and other illicit opioid use.

**What is added by this report?**

Rates of deaths involving heroin increased in all U.S. Census regions from 2006 to 2015. The increase appears to be driven in part by increases in the heroin supply after 2010 and by the introduction of illicitly manufactured fentanyl (IMF), a synthetic opioid, into the heroin market. Deaths involving both heroin and synthetic opioids increased sharply after 2013. The largest increases were in regions where white powder heroin is primarily used. Deaths involving synthetic opioids without heroin also increased sharply after 2013, indicating emergence of synthetic products without heroin or mixing of IMF into other drugs, including cocaine.

**What are the implications for public health practice?**

Changes in the supply and potency of illicit drug products can substantially contribute to increases in overdose deaths regardless of rates of opioid misuse. With continued increases in the heroin and synthetic opioid supply and deaths in the context of prescription opioid misuse, sustained, targeted, and multisectoral responses to the opioid overdose epidemic are needed, including timely surveillance, safer opioid prescribing, education on opioid overdose and naloxone, linkage and access to treatment, leveraging of community-based services, and collaboration between public health and public safety agencies.

## Footnotes

* Death data are from CDC WONDER (https://wonder.cdc.gov/). “Synthetic opioids excluding methadone” is a defined cause of death category.

† *Northeast:* Connecticut, Maine, Massachusetts, New Hampshire, New Jersey, New York, Pennsylvania, and Vermont; *Midwest:* Illinois, Indiana, Iowa, Kansas, Michigan, Minnesota, Missouri, Nebraska, North Dakota, Ohio, Rhode Island, South Dakota, and Wisconsin; *South:* Alabama, Arkansas, Delaware, District of Columbia, Florida, Georgia, Kentucky, Louisiana, Maryland, Mississippi, North Carolina, Oklahoma, South Carolina, Tennessee, Texas, Virginia, and West Virginia; *West:* Alaska, Arizona, California, Colorado, Hawaii, Idaho, Montana, Nevada, New Mexico, Oregon, Utah, Washington, and Wyoming.

§ The Drug Enforcement Administration’s National Forensic Laboratory Information System (NFLIS) estimates of drug exhibits are calculated using the National Estimates Based on All Reports method, which incorporates weighting to account for nonsampled laboratories. https://www.nflis.deadiversion.usdoj.gov/DesktopModules/ReportDownloads/Reports/NFLIS2015AR.pdf .

¶ DEA’s NFLIS collects drug identification results from reports submitted to state and local and national laboratories. https://www.deadiversion.usdoj.gov/nflis/. Drug report estimates are calculated from state and local laboratory reports and were obtained on July 6, 2017.

** Suicides and homicides were excluded because the focus was on unintentional overdoses. Trends in unintentional overdose deaths are expected to be different from those of intentional poisoning deaths. During 2006–2015, only 1.1% and 9.1% of deaths involving heroin or synthetic opioids were categorized as homicides or suicides, respectively (https://wonder.cdc.gov/). Undetermined intent overdoses were included because they might have been unintentional; only 7% of analyzed deaths were categorized as undetermined.

†† “Deaths involving heroin without synthetic opioids” and “deaths involving synthetic opioids without heroin” exclude deaths with synthetic opioids and heroin, respectively, but other substances could have contributed to the deaths.

§§ Fentanyl-related overdose deaths were estimated to have increased by 2,295 from 1,905 in 2013 to 4,200 in 2014 (https://www.cdc.gov/nchs/data/nvsr/nvsr65/nvsr65_10.pdf). This accounts for approximately 94% of the increase in deaths involving synthetic opioid excluding methadone from 3,105 to 5,544.

¶¶ The National Survey on Drug Use and Health collects data on prescription pain reliever use. Although survey respondents can write in nonopioid pain relievers, all pain relievers specifically asked about are opioids. Therefore, misuse of pain relievers is a close approximation of misuse of prescription opioid pain relievers. Misuse of pain relievers was defined as any use in a way not directed by a doctor. https://www.samhsa.gov/data/sites/default/files/NSDUH-DetTabs-2015/NSDUH-DetTabs-2015/NSDUH-DetTabs-2015.pdf.

*** https://www.samhsa.gov/data/sites/default/files/NSDUH-FFR2-2015/NSDUH-FFR2-2015.htm.

††† https://emergency.cdc.gov/han/han00384.asp.

¶¶¶ https://emergency.cdc.gov/han/han00395.asp.

**** https://www.cdc.gov/drugoverdose/prescribing/guideline.html.

†††† https://aspe.hhs.gov/system/files/pdf/107956/ib_OpioidInitiative.pdf.

§§§§ https://www.ncbi.nlm.nih.gov/pmc/articles/PMC2955973/.
